# Machine‐learning prediction of postoperative complications after high tibial osteotomy for canine cranial cruciate ligament disease

**DOI:** 10.1111/vsu.70007

**Published:** 2025-08-29

**Authors:** Daniel Low, Rhys Treharne, Scott Rutherford

**Affiliations:** ^1^ frank. Pet Surgeons Leeds UK; ^2^ Swift Referrals Wetherby UK

## Abstract

**Objective:**

The aim of this study was to develop and internally validate a machine‐learning algorithm, PROSPECT (Predicting Risk Of Surgical complications aftEr CCWO and TPLO), using clinical variables to predict postoperative complications in dogs undergoing high tibial osteotomy for cranial cruciate ligament disease (CrCLD).

**Study design:**

Retrospective multivariable prediction model development.

**Sample population:**

Stifles (*n* = 670) and dogs (*n* = 555).

**Methods:**

Complication data with a minimum follow up of 28 days were collected. Clinical variables were preprocessed for machine learning and interaction features were engineered. A multioutput eXtreme Gradient Boosting model was trained on 80% of the sample to predict minor, surgical, and medical complications independently. The trained PROSPECT model was then tested on the independent test set. Model performance was evaluated qualitatively and quantitatively.

**Results:**

Complications occurred in 134/670 (20.0%) stifles, with 50 (7.5%) minor complications, 69 (10.3%) surgical complications, and 26 (3.4%) medical complications. The PROSPECT model achieved Brier scores and accuracies of 0.06379 ± 0.009100 and 91.9% for minor complications, 0.05481 ± 0.008589 and 92.3% for surgical complications, and 0.04102 ± 0.008194 and 94.3% for medical complications.

**Conclusion:**

The PROSPECT model can predict postoperative complications accurately and in a probabilistic fashion following high tibial osteotomy for CrCLD.

**Clinical significance:**

Machine learning may facilitate an individualized approach to risk management with the potential to enhance patient safety and promote safer surgery.

**Abbreviations**
AUC: area under the receiver operating characteristic
CCWO: cranial closing wedgeostectomy
CrCLD: cranial cruciate ligament disease
CV: coefficient ofvariation
IQR: interquartile range
NPV: negative predictive value
PPV: positive predictive value
PROSPECT: Predicting Risk Of Surgical complications aftEr CCWO and TPLO
PPV: positive predictive value
SD: standard deviation
TPLO: tibial plateau levelling osteotomy
TRIPOD: Transparent Reporting of a multivariable prediction model for Individual Prognosis or Diagnosis
XGBoost: eXtreme Gradient Boosting

## 1 INTRODUCTION

Cranial cruciate ligament disease (CrCLD) is one of the most common causes of pelvic limb lameness in dogs,^1^, ^2^ with an estimated prevalence of 0.56% to 2.55%.^2^, ^3^ Tibial plateau leveling osteotomy (TPLO) and cranial closing wedge ostectomy (CCWO) are surgical techniques to alter the tibial plateau angle and stabilize the cranial cruciate deficient stifle joint.^4^, ^5^, ^6^, ^7^ These techniques are associated with good outcomes^8^, ^9^ but postoperative complication rates between 9.7% and 34% have been reported.^4^, ^10^, ^11^, ^12^, ^13^, ^14^, ^15^ Postoperative complications lead to patient morbidity, may require additional medical or surgical treatment,^4^, ^12^ and are also associated with increased veterinary health‐care costs.^16^

Risk factors for postoperative complications after high tibial osteotomy for CrCLD have been investigated extensively in retrospective and prospective studies.^4^, ^7^, ^10^, ^11^, ^12^, ^13^, ^14^, ^15^, ^17^, ^18^, ^19^ Traditional regression‐based analyses provide an understanding of risk factors at the population level by estimating the average effect of each variable via odds ratios. However, the generalizability of population‐level odds ratios to individual patients is imperfect.^20^ Differences in study populations, methodologies, and varying degrees of bias and confounding factors mean that previously reported risk factors and complication rates do not always apply to an individual dog.^21^, ^22^ Model‐driven approaches such as with multivariable logistic regression also make underlying assumptions about associations between model inputs and outputs,^23^ and may not adequately capture real‐world complex interactions between investigated variables and outcomes.^24^, ^25^ Even when trained on large quantities of real‐world data these models are not immediately generalizable to external patient populations.^26^, ^27^

Machine‐learning algorithms may predict postoperative complications with a data‐driven approach instead.^28^, ^29^ These algorithms have been investigated as risk prediction tools in humans with medical and surgical conditions^30^, ^31^ and have been shown to produce superior predictive models that retain their robustness despite changes in underlying patient populations.^26^ Machine‐learning algorithms have been investigated as postoperative risk‐prediction tools in total hip and knee arthroplasty in man^32^, ^33^ and in dogs with severe acute spinal cord injury secondary to intervertebral disc extrusion^34^ but they remain inadequately investigated in veterinary orthopedic surgery.

A machine‐learning algorithm to predict postoperative complication risk in high tibial osteotomy for CrCLD would be a novel assistive tool for surgical decision making, providing information about an individual dog's risk that was previously unavailable to veterinary orthopedic surgeons. This would allow a precise approach to preoperative patient selection, client counseling, targeted risk reduction strategies, and targeted postoperative surveillance.

This study aimed to develop and internally validate a machine‐learning algorithm (PROSPECT: Predicting Risk of Surgical Complications after CCWO and TPLO) for predicting postoperative complications following high tibial osteotomy for CrCLD using retrospective clinical data. A secondary aim was to derive model performance metrics, calibration plots, and feature importance values to illustrate the PROSPECT model's decision making and its potential application in clinical practice.

## 2 MATERIAL AND METHODS

This study was reported in accordance with the Transparent Reporting of a Multivariable Prediction model for Individual Prognosis or Diagnosis (TRIPOD) guidelines (Supporting Information, File S1).^35^ All data in this study were obtained with written client consent granting permission for the anonymized use of patient data for research purposes.

### 2.1 Data extraction and outcome definition

Retrospective data extraction was performed using a hospital electronic health records search (EasyVet; VetZ GmbH, Germany). Records from 2018 to 2023 were searched for “CrCLD” and “TPLO” or “CCWO.” Dogs were included if they had a diagnosis of CrCLD and underwent either TPLO or CCWO. Records were excluded if short‐term follow‐up data were unavailable; this was defined as a 28‐day minimum follow‐up period. Records were also excluded if surgery on the stifle or proximal tibia had been performed previously. Data collected included patient age at the time of surgery, breed, bodyweight, sex and neuter status, attending surgeon, operated limb, bilaterality, surgical technique, surgical implants used, performance of stifle arthrotomy, performance of meniscal surgery, intraoperative complications, all postoperative complications, and time to follow up.

Dogs were assigned into cohorts based on whether they developed a postoperative complication. Postoperative complications were defined as minor, surgical, and medical complications, as described in the literature.^36^ Surgical site infections were diagnosed according to standard criteria.^37^ Complications were assessed via systematic orthopedic examination and follow‐up radiography of the stifle, which was routinely requested 4 to 8 weeks postoperatively. In the event of a complication, whether before or after routine follow up, patients were re‐examined and treated appropriately, as often as necessary until resolution of the complication.

### 2.2 Surgical procedure

Surgical procedures were performed by board‐certified surgeons (ECVS or ACVS), residents under direct supervision, or nonboarded clinicians. Preoperative stifle radiography was performed for diagnostic purposes and surgical planning immediately prior to surgery. Perioperative cefuroxime or amoxicillin‐clavulanate 20 mg/kg was administered 30 minutes preoperatively and repeated every 90 minutes intraoperatively. Antibiotics were not routinely continued into the postoperative period and were only prescribed exceptionally at the discretion of the attending surgeon. Standard TPLO without a jig or modified CCWO^7^ was performed. Neither routine stifle joint inspection nor meniscal treatment was performed and only a selected minority of stifles underwent miniarthrotomy and meniscal treatment. Postoperative radiography was performed to confirm satisfactory implant placement. Dogs were hospitalized for 24–48 h postoperatively for opioid analgesia and discharged with a nonsteroidal anti‐inflammatory drug for 2 to 8 weeks. Two weeks of restricted activity was recommended and a gradually increasing regimen of lead‐only walks was introduced from week 3 onwards.

### 2.3 Predictive modeling

Each operated stifle corresponded to one unique record. Patients who underwent single‐stage bilateral high tibial osteotomy or staged bilateral high tibial osteotomy had a unique record for each operated stifle. Twelve preoperative variables were selected based on retrospective data availability and for potential clinical utility, as all variables are readily available in clinical practice without the need for nonroutine diagnostic procedures. Continuous variables included patient age at the time of surgery and bodyweight. Categorical variables included patient breed, sex and neuter status, attending surgeon, operated limb, bilaterality, surgical technique, surgical implants used, performance of stifle arthrotomy, performance of meniscal surgery, and presence of intraoperative complications. Surgical implants were defined categorically as Synthes TPLO plate (Johnson & Johnson MedTech; New Jersey, USA) only, String of Pearls TPLO plate (Orthomed; United Kingdom) only, or “other” if adjunctive plate and screw fixation was used. Stifle arthrotomy, meniscal surgery, and intraoperative complications were defined as binary variables.

Univariate analysis was performed using the *χ* ^2^ test for categorical variables and univariate regression for continuous variables. Significant risk factors for postoperative complications are reported as odds ratios and 95% confidence intervals (CI).

The dataset was randomly split into training and testing sets in an 80:20 ratio utilizing a pseudorandom split function with a consistent seed. Continuous variables were standardized using a standard scaler, and categorical variables underwent one‐hot encoding. Polynomial feature expansion was applied, generating engineered second‐degree interaction features without including squared terms. Class imbalance was addressed using the synthetic minority oversampling technique for nominal and continuous data. Preprocessing generated 3240 final features for model training. To evaluate the influence of surgeon variables on model performance, a secondary model was constructed using the same pipeline, with all surgeon‐related variables removed.

A multioutput eXtreme Gradient Boosting (XGBoost) model was trained and tuned on the data using automated Bayesian hyperparameter tuning, with mean squared error (Brier score) as the loss‐minimizing metric. The model generated three independent probability scores between 0 and 1, corresponding to each class of postoperative complication. To assess model generalizability and account for sampling variability, the entire modeling pipeline, including resampling, preprocessing, training, and evaluation, was repeated across 100 independent runs using different random seeds. To assess the reproducibility of model predictions across different training instances, a set of 50 dogs was held out as a fixed validation cohort. For each run, the data were split into training and test sets with stratification by complication class, and internal validation was performed on the independent test set. Internal validation was performed on an independent test set, with predictive performance primarily assessed using the Brier score. To evaluate classification performance of each generated probability score, subset accuracy, sensitivity, specificity, positive predictive value (PPV), negative predictive value (NPV), and the coefficient of variation (CV) were calculated per run and reported summarily across the 100 runs. For classification performance calculation, probabilistic predictions were thresholded at 0.5, with predictions less than 0.5 indicative of no postoperative complications and predictions greater than 0.5 indicative of a postoperative complication. The area under the receiver operating characteristic curve (AUC) was calculated as the area under the curve plotting the true positive rate against the false positive rate across all possible threshold values. Model calibration across the 100 runs was assessed using calibration curves and probability distribution plots. Brier scores, calibration curves, and probability distribution plots of the full model were plotted against the model with surgeon variables removed. Paired *t*‐tests were used to compare Brier scores from the full model against the model with surgeon variables removed, across 100 runs. Class‐level average CVs were then used to evaluate the consistency of probabilistic outputs across model iterations. Feature importance values were obtained directly from XGBoost, and permutation importance was used to assess the directionality of feature contributions. Feature importance plots were generated to visualize the most influential features and their impact on model predictions. Data normality was assessed with the Shapiro–Wilk test. Nonparametric data are expressed as median and interquartile range (IQR) and parametric data expressed as mean and standard deviation (SD). Statistical significance was defined as *p* < .05. All statistical analysis, predictive modeling, and data visualization were performed with *pandas 2.2.2*, *numpy 1.26.4*, *scipy 1.14.1*, *scikit‐learn 1.6.1*, *imblearn 0.13.0*, *xgboost 2.1.4*, *optuna 4.2.1*, *seaborn 0.13.2*, and *matplotlib 3.10.0* in Python version 3.11.13.^38^, ^39^, ^40^, ^41^, ^42^, ^43^, ^44^, ^45^, ^46^

## 3 RESULTS

The medical record search yielded 697 consecutive stifles from 581 dogs that underwent high tibial osteotomy for CrCLD between January 2018 and December 2023. Fifteen stifles (15 dogs) were excluded due to prior stifle or tibial surgery. Seven stifles (six dogs) were excluded due to death before follow up. Five stifles (five dogs) were excluded due to failure to attend follow up. Overall, 670 stifles from 555 dogs met inclusion criteria.

The median age at the time of surgery was 6.25 years (IQR: 4.25–8.5 years) and the median bodyweight was 28.2 kg (IQR: 18.7–36.8 kg). The most common breeds included were crossbred dogs (*n* = 142), Labrador retrievers (*n* = 83), golden retrievers (*n* = 42) and English Springer spaniels (*n* = 29). Neutered female dogs were overrepresented with 281 females (213 neutered) and 274 males (201 neutered). The laterality of the operated limb was equally distributed with 340 left stifles and 330 right stifles operated. Thirty‐one dogs (5.6%) had bilateral single‐stage surgery and 84 dogs (15.1%) had bilateral staged surgery. Cranial closing wedge ostectomy was performed in 269 stifles (40.1%) and TPLO performed in the remaining 401 stifles (59.9%). Stabilization was achieved with Synthes TPLO implants in 462 stifles (69.0%), SOP TPLO implants in 170 stifles (25.4%), and adjunctive fixation in 38 stifles (5.7%). Stifle joint inspection was performed in a minority of selected stifles (*n* = 80, 11.9%) and, of these, 63 stifles (9.4%) underwent meniscectomy or meniscal release.

There were intraoperative complications in seven stifles. Five of these were hardware complications such as drill bit or Kirschner wire breakage and the remaining two were hemorrhage from the cranial tibial artery.

Postoperative follow‐up data were obtained at a median of 49 days (IQR: 43–110 days). Postoperative complications occurred at a median of 23.5 days (IQR: 10–50 days), with the distribution of complications skewed towards the first 6 weeks after operation (Supporting Information, File S2). Complications were noted in 134 stifles (20.0%). Of these, 50 (7.5%) were minor complications, 69 (10.3%) were surgical complications, and 26 (3.4%) were medical complications, with 15 stifles having more than one postoperative complication. Minor complications included seroma formation (*n* = 20), fibular fracture (*n* = 12), implant‐related complications not requiring further treatment (*n* = 11), tibial tuberosity fracture (*n* = 4), and minor incisional complications (*n* = 3). Surgical complications included implant‐associated surgical site infection (*n* = 33), late meniscal injury (n = 20), wound dehiscence (*n* = 16), and implant failure (*n* = 5). Medical complications all consisted of superficial incisional surgical site infections (*n* = 26).

Univariable analysis showed that bodyweight (*p* = .002, OR = 1.02, 95% CI: 1.01–1.04), the bulldog breed (*p* = .011, crude OR = 3.06, 95% CI: 1.30–7.22), the German shepherd breed (*p* = .01, OR = 4.30, 95% CI: 1.41–13.1), the Rottweiler breed (*p* < .001, OR = 6.55, 95% CI: 2.51–17.1), and intact male status (*p* < .001, OR = 2.67, 95% CI: 1.54–4.63) were associated with an increased risk of postoperative complications. The Labrador retriever breed (*p* = .013, OR = 0.33, 95% CI: 0.14–0.79) was associated with a decreased risk of postoperative complications.

**FIGURE 1:**
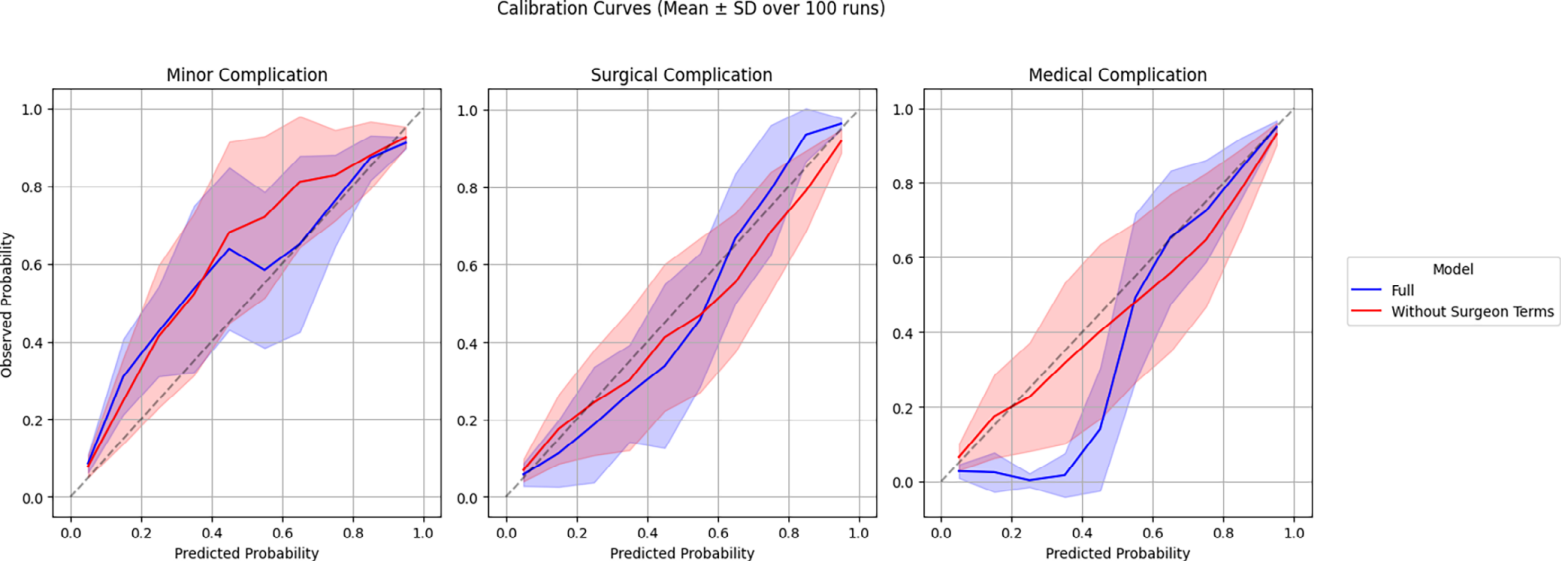
Calibration curves and 95% confidence intervals of the full model and the model without surgeon terms, by class of surgical complication over 100 runs, illustrating model calibration across the range of predicted probabilities. The dotted line represents perfect calibration.

Bayesian hyperparameter optimization yielded the following PROSPECT model parameters: 400 trees, maximum tree depth of three, learning rate of 0.16, subsample ratio of 0.80, and column subsample ratio per tree of 0.92. Calibration curves across 100 runs showed moderate to excellent model calibration (Figure 1). The model's predictions were generally well calibrated except for the full model's prediction of medical complications, where moderate overestimation of complication risk occurred at the lower end of the spectrum. There was mild to moderate overestimation or underestimation of postoperative complications within the centers of the curves, indicating good, but not excellent, certainty with regards to predicting the occurrence of postoperative complications. The full model demonstrated lower standard deviation and was generally better calibrated than the model without surgeon terms.

**FIGURE 2:**
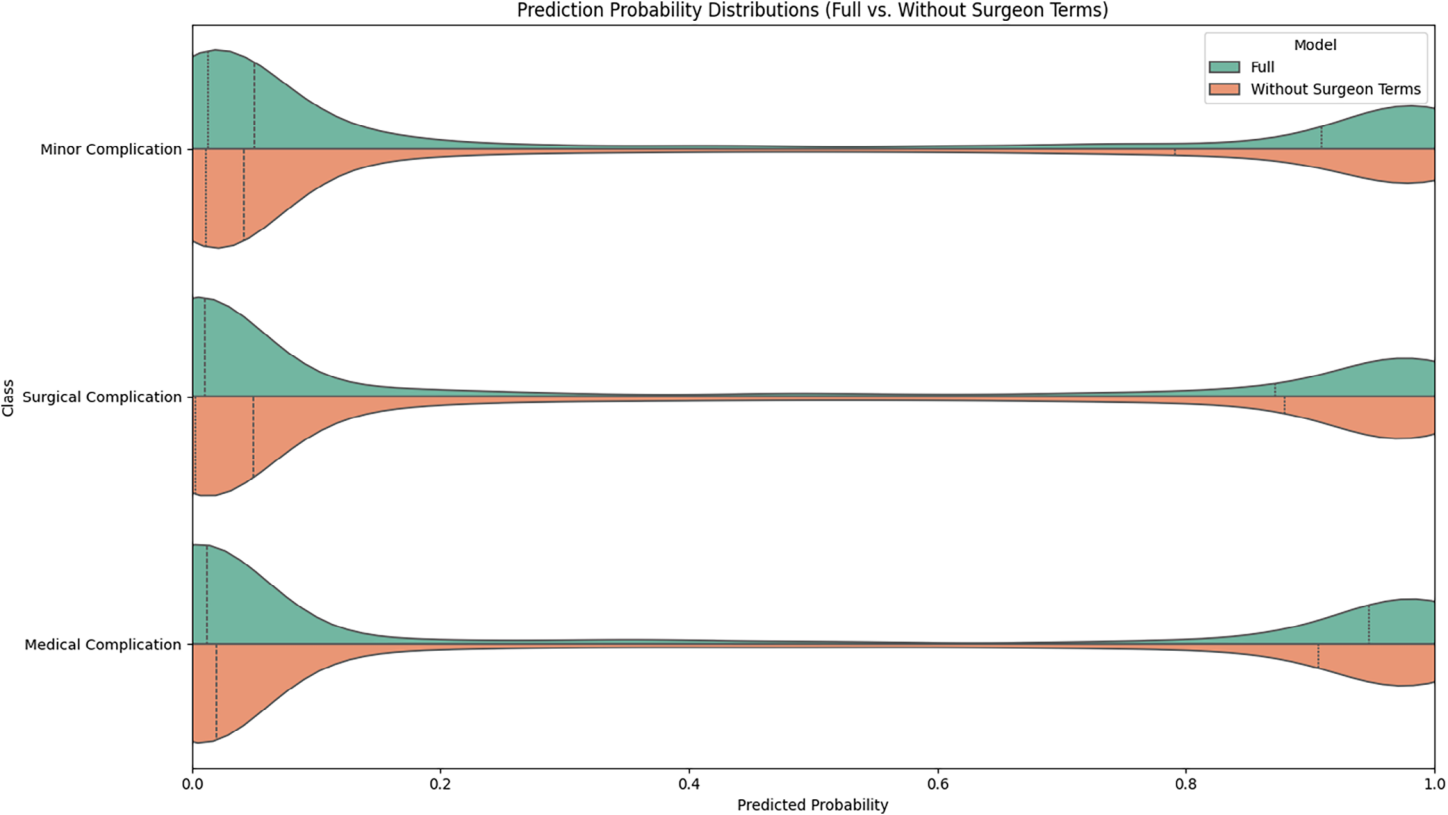
Violin plots representing distribution of predictions of the full model and the model without surgeon terms, by class of surgical complication over 100 runs, illustrating the spread and density of model predictions. Wider sections indicate a higher density of predictions at a given probability, and narrower sections indicate fewer predictions. Dotted lines denote quartiles.

Figure 2 shows prediction probability distributions for the PROSPECT model and the model without surgeon terms. Both display a concentration of predicted probabilities at the extremes, reflecting high certainty in most cases, whereas a broader spread in the midrange of the violin plot indicates greater uncertainty for moderate probabilities.

**FIGURE 3:**
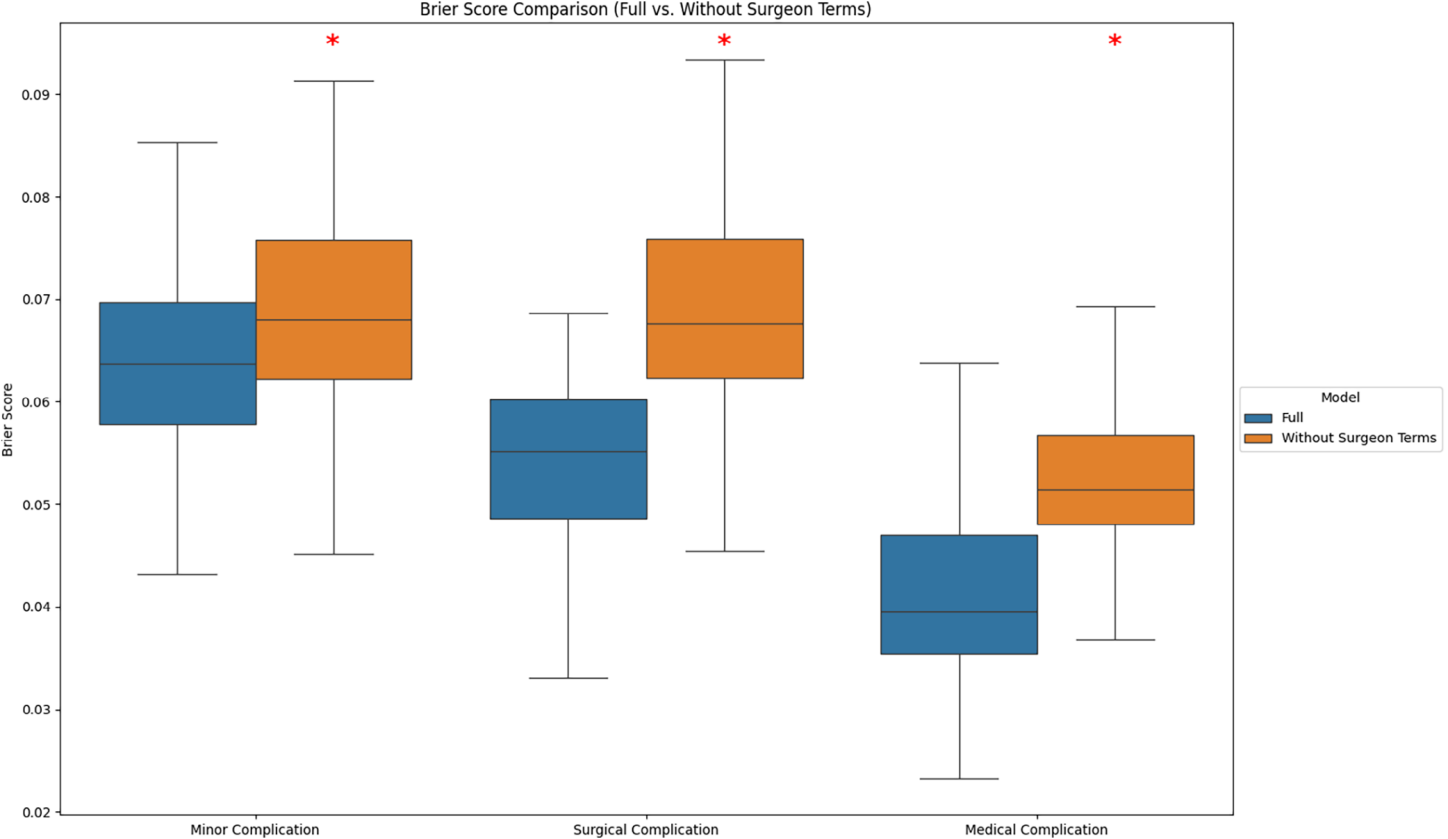
Box‐and‐whisker plots representing Brier scores of the full model and the model without surgeon terms, by class of surgical complication over 100 runs, illustrating the predictive performance of each model across each class of complication. Statistical comparisons were performed using paired *t*‐tests.

The PROSPECT model achieved an average weighted Brier score of 0.06965 ± 0.005381 across all classes of complications on the independent test set across 100 runs, with a subset Brier score of 0.06379 ± 0.009100 for minor complications, 0.05481 ± 0.008589 for surgical complications, and 0.04102 ± 0.008194 for medical complications. The model without surgeon terms achieved subset Brier scores of 0.06864 ± 0.009339 for minor complications, 0.06876 ± 0.01070 for surgical complications, and 0.05244 ± 0.007979 for medical complications, which were different from those of the full model (Figure 3; all *p* < .001). Table 1 shows the remaining model metrics by class of complications. Coefficients of variation of predicted probabilities across 100 runs applied to a fixed set of 50 dogs were 0.47 (MinorC), 0.59 (SurgC), and 0.66 (MedC), indicating moderate intermodel variability.

**TABLE 1:** Performance metrics of the PROSPECT model's predictions by class of surgical complication, represented as means and standard deviations across 100 runs.

Complication class	AUC	Subset accuracy	Sensitivity	Specificity	PPV	NPV
Minor	0.9578 ± 0.01184	0.9193 ± 0.01172	0.8360 ± 0.03232	0.9609 ± 0.0117	0.9151 ± 0.02272	0.9217 ± 0.01401
Surgical	0.9661 ± 0.009348	0.9230 ± 0.01322	0.8955 ± 0.02956	0.9366 ± 0.01493	0.8770 ± 0.02526	0.9474 ± 0.01416
Medical	0.9819 ± 0.005606	0.9425 ± 0.01297	0.9252 ± 0.02733	0.9512 ± 0.01471	0.9054 ± 0.02564	0.9623 ± 0.01335

Abbreviations: AUC, area under the receiver operating characteristic curve; NPV, negative predictive value; PPV, positive predictive value.

**FIGURE 4:**
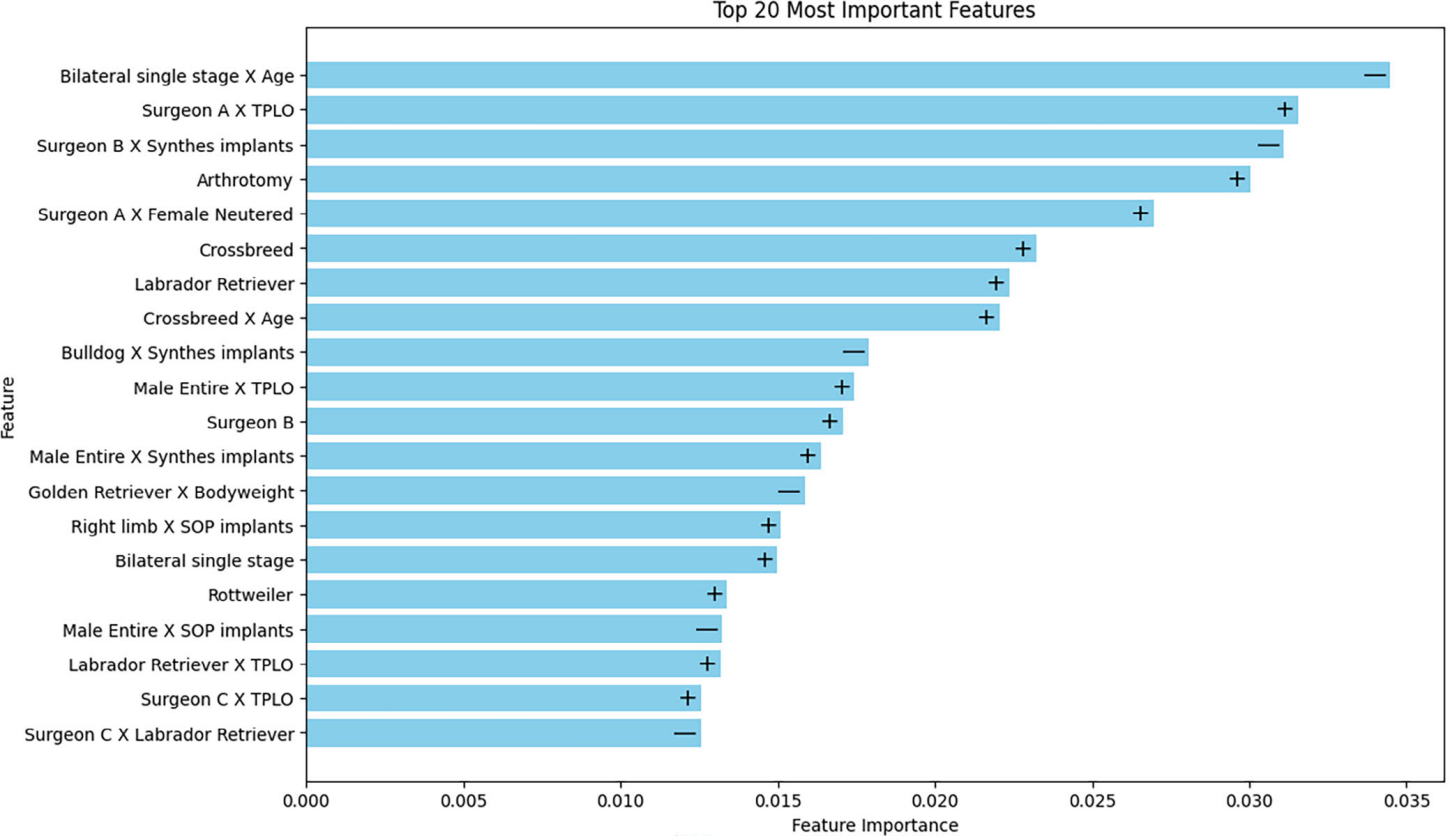
Feature importance plot illustrating the 20 most important features contributing to the PROSPECT model's predictions, with the directionality of each feature's impact denoted by + (positive impact) and − (negative impact).

Figure 4 shows the features with the greatest impact on the PROSPECT model's decisions and their directionality of impact. The top three features were engineered interaction features and included clinical variables such as the surgeon, implant type, surgical technique, performance of bilateral single stage surgery, and patient age. Fourteen out of the 20 most important features were engineered interaction features. The directionality of the contribution of each feature was mixed, with six features reducing the PROSPECT model's probabilistic prediction towards a postoperative complication and 13 features increasing it. Six features included the surgeon as a variable. The majority were engineered interaction features and their impact was bidirectional. Fifteen out of the 20 most important features were related to the surgeon, surgical technique, implant type, or other modifiable risk factors.

## 4 DISCUSSION

This study shows that the PROSPECT machine‐learning model can accurately identify postoperative complications in individual dogs after high tibial osteotomy for CrCLD. It applied a multioutput machine‐learning approach, which successfully identified complications in the majority of operated stifles. Notably, its findings suggest that postoperative complications are not driven by simple associations with individual clinical variables; instead, complex interactions between multiple factors drive the PROSPECT model's predictions.

Machine‐learning algorithms have been explored as diagnostic and prognostic tools in veterinary radiology, internal medicine, pharmacology, and public health,^47^, ^48^, ^49^, ^50^, ^51^, ^52^ and for predicting neurosurgical outcomes using electronic healthcare data.^34^

This study is the first report of applied machine learning in veterinary orthopedic surgery and the first report of multioutput probabilistic postoperative risk prediction in veterinary surgery. Multioutput machine‐learning algorithms have been investigated in a range of inpatient surgical procedures in man^53^ and this study demonstrates a workflow in a veterinary population whereby three distinct classes of postoperative complications may be predicted. Despite the fact that high tibial osteotomy is typically a clean surgery performed electively in a healthy patient population without comorbidities, the postoperative complication rate ranged from 9.7% to 34%.^4^, ^10^, ^11^, ^12^, ^13^, ^14^, ^15^ Minor, surgical, and medical complications differ in terms of their impact on patient morbidity, the nature of additional treatment required (if any), and the cost of additional treatment. Postoperative risk prediction and preoperative client counseling are currently limited by the broad nature of risk prediction, with figures typically drawn from population‐level studies and poorly tailored to individual dogs. Furthermore, traditional statistical models are limited in that relationships between variables are assumed prior to analysis,^28^ and this may partly explain the differences in reported risk factors between studies.

Machine‐learning models identify patterns within datasets without relying on prior assumptions, and are able to capture complex real‐world associations more effectively.^28^ In this study, an XGBoost model was used as it had been shown to excel as a general purpose classifier,^44^, ^53^ and in veterinary neurosurgical outcome prediction specifically.^34^ The PROSPECT model relied heavily on feature engineering to achieve the predictive performance reported. Feature engineering has been applied previously in a similar tree‐based model to predict postoperative complications across a wide range of surgical procedures in man.^54^ Beginning with 12 continuous and categorical clinical variables, interaction features were engineered to produce over 3000 features for model training. Interaction features introduce dependencies between two or more raw variables, potentially uncovering complex relationships that may not be evident when analyzing variables independently.^55^

Machine learning is integral to feature engineering, as high‐dimensional data necessitates explicit or implicit feature selection. The XGBoost base model is well suited for this task, as it inherently performs feature selection during training.^44^ The feature importance plot demonstrates that the majority of predictive signals in the PROSPECT model were derived from engineered features, reinforcing that postoperative complications after high tibial osteotomy are driven by complex, nonlinear relationships.

Univariable statistical analysis in this study showed that bodyweight and certain breeds were associated with an increased risk of postoperative complications, which aligns with previous reports using similar statistical methods.^4^, ^12^, ^15^ The Labrador retriever and Rottweiler breeds were found to be protective against and predisposing to postoperative complications, respectively, on univariable analysis. This was consistent with findings in previous reports.^4^, ^12^ However, it is noteworthy that few of these univariable associations, apart from the Rottweiler breed, were deemed important in the PROSPECT model's decision making. This again suggests that traditional statistical analyses are limited when attempting to model real‐world interactions.

The PROSPECT model did not rely heavily on any single raw clinical variable to produce probabilistic predictions, echoing findings from studies where tree‐based models utilize multiple weak predictors to produce a stronger, more robust predictor.^34^ The majority of the top 20 most important features were engineered interaction features, suggesting that risk factors are not universal and are instead dynamic and context specific. In other words, the interactions between clinical variables in the context of individual patients are more indicative of postoperative outcomes than any single, isolated factor. For example, previous studies report arthrotomy as a risk factor for postoperative complications,^12^, ^13^ but this study shows that this association may not hold in every instance. From the feature importance plots, arthrotomy increased the risk of postoperative complications as a raw clinical variable but not in the case of arthrotomy‐containing interaction features. Machine‐learning models may therefore be superior in being able to dynamically predict postoperative risk in the context of an individual dog. The PROSPECT model, in capturing complex, nonlinear relationships that traditional statistical methods may miss, provides a more accurate and individualized approach to predicting surgical complications.

Raw performance metrics from the PROSPECT model indicate that its predictions were both qualitatively accurate and quantitatively precise. In approximately 90% of cases, the predicted probability aligned with the ground truth (<0.5 in the absence of complications and >0.5 in the presence of complications). Model calibration was generally good at both extremes of the probability spectrum, with mild to moderate deviations in the midrange.

This suggests that the PROSPECT model reliably identified dogs at very low and very high risk of postoperative complications, whereas predictions in the intermediate range were associated with greater uncertainty. Unlike binary classification machine‐learning models, the PROSPECT model generates probabilistic predictions, reflecting degrees of certainty about postoperative outcomes. This is particularly valuable in clinical settings where decision making is inherently uncertain. Predictions near 0 or 1 indicate strong confidence in the absence or presence of complications, respectively, whereas intermediate probabilities highlight cases where outcomes are less certain. Predictions for minor complications were relatively stable across models, whereas surgical complications and medical complications showed higher variation, indicating greater sensitivity of these classes to data resampling or model initialization. This variability reflects the challenge of achieving consistent prediction in limited datasets and suggests that further research is required before clinical deployment.

The feature importance metrics suggest that the attending surgeon and surgical decision making influence the risk of a postoperative complication, and may represent modifiable risk factors in high tibial osteotomy. Variability in reported complication rates across studies is, at least in part, likely to be attributable to surgeon‐related factors. For example, a large case series of dogs undergoing TPLO by a single experienced surgeon reported lower complication rates compared to other studies, reinforcing the importance of surgical experience.^4^

Machine‐learning algorithms are often criticized as black‐box models with limited interpretability,^56^ so the feature importance plot provides a visual and quantitative representation of how the PROSPECT model weighs individual features in generating predictions. However, the inherent complexity of machine‐learning makes it challenging to derive immediately actionable recommendations for clinical practice. While surgeon‐related factors, such as surgical technique and implant selection, are modifiable, the extent to which they can be optimized to reduce complication risk remains unknown.

An individual dog's risk is likely to be context dependent, meaning that isolated feature importance values may not fully capture the relationships between clinical variables. The heavy weighting of surgeon‐related factors may limit the PROSPECT model's immediate generalizability to other surgeons and populations, as the model has not been trained on surgical practices beyond those represented in the dataset. The magnitude of Brier score differences between the full model and the model without surgeon terms was small and whether it translates to a meaningful clinical difference is unknown. External validation on new surgeons or institutions will likely require a period of model adaptation or a learning curve as the algorithm encounters unfamiliar decision‐making patterns. Further research is therefore needed to better understand how these factors interact and contribute to postoperative complication risk following high tibial osteotomy for CrCLD, and to what extent these factors differ between surgeons and institutions.

A machine‐learning approach for postoperative risk management would have an impact on surgical decision making related to high tibial osteotomy for CrCLD. Surgeons can currently only estimate a dog's risk of postoperative complications based on available evidence and prior experience. Incorporating the PROSPECT model into clinical decision making can enhance individualized patient care. Preoperatively, this tool will allow for the identification of patients at high risk of postoperative complications, creating opportunities to manage client expectations and to implement targeted risk reduction strategies or targeted postoperative surveillance. In certain cases, it may also be appropriate to avoid surgery altogether. Postoperatively, a dog predicted to have a low probability of complications (<10%) may be discharged with routine postoperative care, whereas a dog with a high probability (>90%) may benefit from proactive intervention or surveillance. Dogs with midrange probability scores (e.g., 40% to 60%) indicate situations where the PROSPECT model is uncertain, and clinical judgment should take precedence.

Outside of clinical practice, the PROSPECT model may also be useful in research, for conducting focused clinical trials in high‐risk patient populations. For example, with high tibial osteotomy for CrCLD, the effect of postoperative antibiotics on surgical site infection rates remains uncertain.^39^ The PROSPECT model may identify dogs at high risk of surgical site infection for a randomized controlled trial, decreasing both the number of individuals needing to be recruited and the blanket use of postoperative antibiotics.

The follow‐up methodology in this study minimized exclusion bias in that fewer than 4% of stifles were excluded from the sample population for any reason. Previous studies have reported loss to follow up varying between 64% and 93%,^7^, ^13^, ^15^ which has been shown to lead to the underestimation of postoperative complications.^57^ The majority of dogs returned for systematic orthopedic re‐examination and follow‐up radiography; hence the degree of underestimation of postoperative complications in the current study is likely to be minimal. However, certain postoperative complications, such as implant‐associated surgical site infections, can occur in a delayed fashion^58^, ^59^ and the future development of delayed complications in this study's population of dogs cannot be ruled out.

Nonetheless, the complication rate reported herein is likely to reflect the true complication rate closely, and thus provides an accurate ground truth for predictive modeling. The postoperative complication rate in this study is within the range of previous reports.

The sample population in this study is similar to previous reports with large‐breed female dogs overrepresented, although this likely also reflects a referral bias with small‐breed dogs underrepresented.^60^

Given this study's preliminary nature, several limitations should be noted. Machine‐learning algorithms require large datasets to allow adequate model generalization and this study was limited by its sample size. Although the sample size was relatively large for a veterinary study, it remains a small sample for machine learning. The clinical data in this study was collected based on retrospective data availability. Anesthetic and surgical time was not consistently available from medical records and therefore not included, but may be potentially useful in future machine‐learning modeling. In other studies, additional variables such as patient comorbidities, American Society of Anesthesiologists grade, anesthetic duration, and biochemical data have been used as inputs for machine‐learning modeling.^26^ The process of feature optimization is empirical^61^ and whether alternative feature engineering techniques may have been beneficial is unknown.

At the authors’ institution, arthrotomy and meniscal surgery are not performed routinely, a practice followed by only a minority of surgeons.^62^, ^63^ In conjunction with model limitations discussed earlier, the immediate external validity of the PROSPECT model may be limited. Future models adapted for specific groups of surgeons and hospitals may be necessary prior to clinical application. Given the limitations mentioned above and the exploratory nature of this study, future research should be directed at gathering large, multi‐institutional datasets for refinement of the PROSPECT model, prior to clinical implementation. Further research into machine‐learning modeling of risk factors may produce clinically actionable conclusions.

In summary, this study demonstrates that a machine‐learning algorithm can accurately predict postoperative complications following high tibial osteotomy for CrCLD. Postoperative risk is influenced by complex, nonlinear, and context‐dependent interactions between clinical variables. Surgeon variables influenced the risk of postoperative complications, which could not be detected on traditional statistical analysis. The PROSPECT model enables an individualized, probabilistic approach to risk management. This shift from deterministic to probabilistic risk assessment represents a meaningful advancement in surgical risk management, with the potential to enhance patient safety and promote safer surgery.

## CONFLICT OF INTEREST STATEMENT

The authors declare no conflicts of interest related to this report.

## Supporting information


File S1. TRIPOD reporting checklist.

**File S2.** Distribution of postoperative complications after tibial plateau leveling osteotomy and cranial closing wedge ostectomy.
