# Supplementary figures and images for: Machine‐learning prediction of postoperative complications after high tibial osteotomy for canine cranial cruciate ligament disease
Source: Vet Surg. 2025 Aug 29;54(7):1286–97. doi: 10.1111/vsu.70007 (PMC12528822; doi:10.1111/vsu.70007)

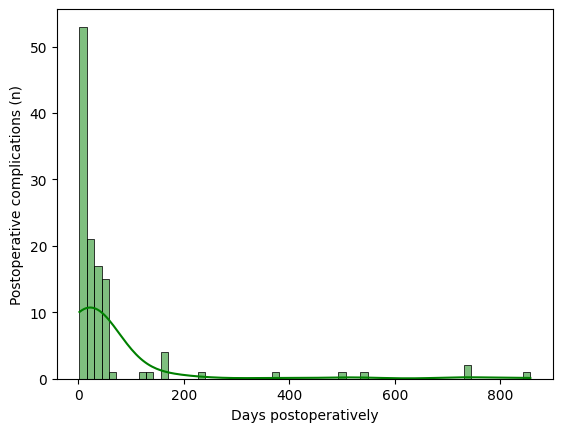

Supplement: Supplementary file 2 — File S2. Distribution of postoperative complications after tibial plateau leveling osteotomy and cranial closing wedge ostectomy. [file VSU-54-1286-s001.png]
